# Supplementary figures and images for: Efficacy of β-lactam/β-lactamase inhibitor combination is linked to WhiB4-mediated changes in redox physiology of Mycobacterium tuberculosis
Source: eLife. 2017 May 26;6:e25624. doi: 10.7554/eLife.25624 (PMC5473688; doi:10.7554/eLife.25624)

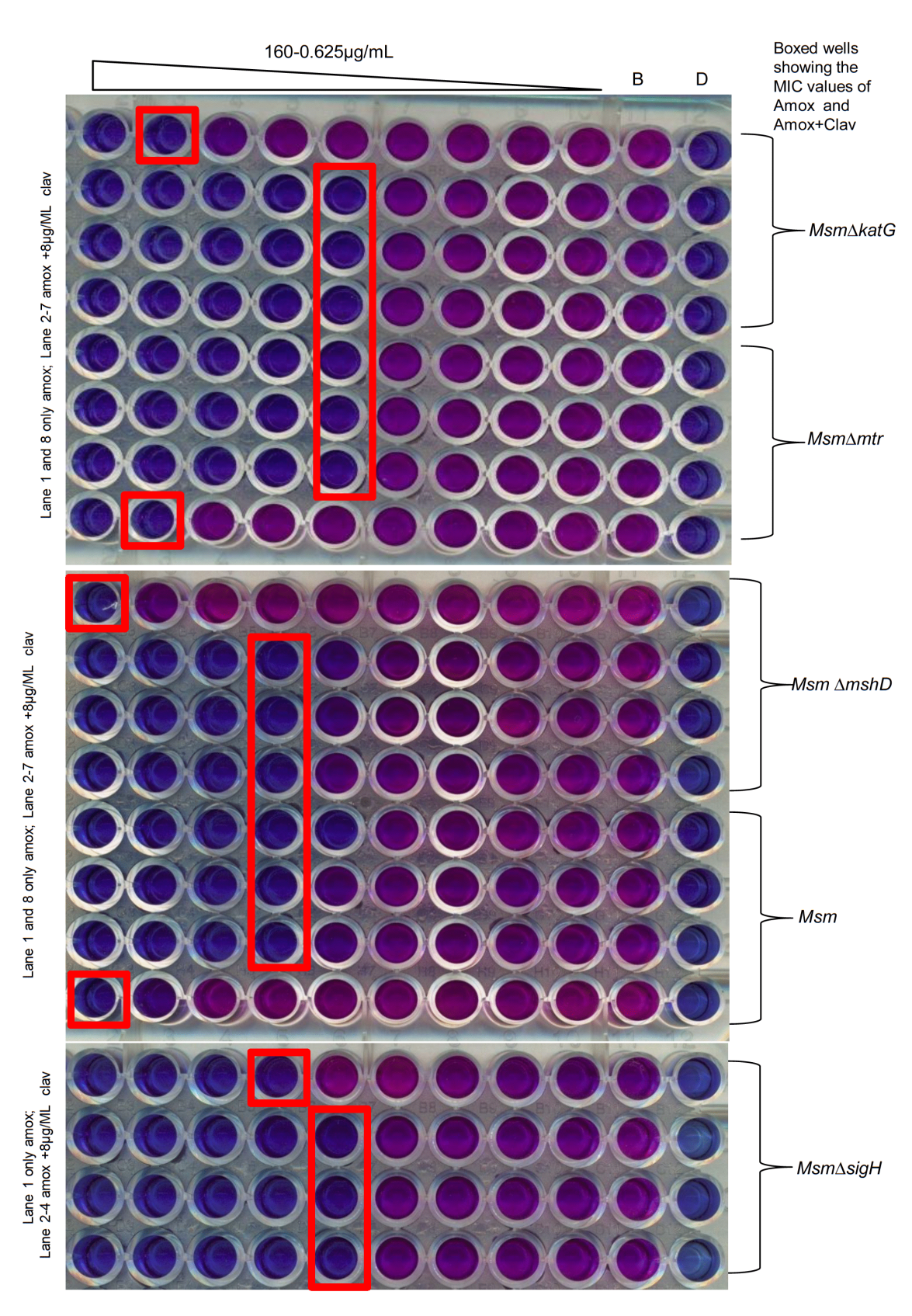

Supplement: Table 1—source data 1. — DOI: http://dx.doi.org/10.7554/eLife.25624.016 [file elife-25624-table1-data1.docx]
